# Dimethyl fumarate alleviate hepatic ischemia–reperfusion injury through suppressing cGAS‐STING signaling

**DOI:** 10.1002/mco2.70077

**Published:** 2025-01-28

**Authors:** Yi Xiong, Jiawen Chen, Kun Li, Wei Liang, Jinwen Song, Xiusheng Qiu, Baoyu Zhang, Dongbo Qiu, Yunfei Qin

**Affiliations:** ^1^ Biotherapy Center The Third Affiliated Hospital of Sun Yat‐sen University Guangzhou Guangdong P. R. China; ^2^ Vaccine Research Institute The Third Affiliated Hospital of Sun Yat‐sen University, Sun Yat‐sen University Guangzhou Guangdong P. R. China; ^3^ Neurosurgery Department The Third Affiliated Hospital of Sun Yat‐sen University Guangzhou Guangdong P. R. China

**Keywords:** cGAS‐STING, Dimethyl fumarate, Hepatic ischemia–reperfusion injury, Innate immunity

## Abstract

Hepatic ischemia–reperfusion (I/R) injury frequently occurs during the perioperative phase of liver surgery. Inappropriate activation of STING signaling can trigger excessive inflammation response to aggravate hepatic I/R injury. Dimethyl fumarate (DMF) is an FDA‐approved immunomodulatory drug used to treat multiple sclerosis and psoriasis due to its notable anti‐inflammation properties. However, the mechanism and targets of DMF in immunomodulation remain unclear. Here, we found that DMF suppresses cGAS‐STING activation induced by HSV‐1, hering testis DNA, and mitochondrial DNA in a variety of cells. DMF significantly reduces hepatic I/R injury and inhibits cGAS‐STING pathway activation in mice. The alleviating effect of DMF on hepatic I/R injury was negligible in STING‐knockout mice. Mechanistically, DMF directly inhibits STING activation via an autophagy‐independent pathway, and the immunocoprecipitation experiment showed that DMF inhibited STING recruitment of downstream TBK1 and IRF3. Our study found that DMF protects liver I/R injury by inhibiting the STING pathway and may be a potential target of this disease.

## Graphical abstract

During hepatic I/R injury, increased mtDNA triggered the activation of cGAS‐STING signaling pathway. DMF can inhibit the interaction between STING and downstream TBK1 or IRF3, block the activation of STING pathway, inhibit the occurrence of inflammation, and protect liver I/R injury.

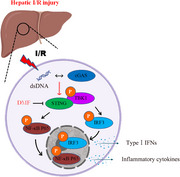

## 1 INTRODUCTION

Hepatic ischemia–reperfusion (I/R) injury is a frequent complication during liver surgery, such as resection and transplantation, and a significant risk factor for post‐transplant rejection and liver failure.^1^, ^2^ At present, there are different opinions on the mechanism of its occurrence and development. Innate immunity, oxidative stress, and metabolic disorders are considered to be the main mechanisms regulating hepatic I/R injury.^3^, ^4^ The cGAS‐STING pathway plays a crucial role in hepatic I/R injury development.^5^, ^6^ Previous research indicates that STING activation exacerbates liver inflammation and hepatic I/R injury by elevating intracellular Ca^2+^ levels and activating GSDMD.^5^ Another study has demonstrated that miR24‐3p inhibits cGAS‐STING signaling, reducing liver inflammation and thereby mitigating hepatic I/R injury.^6^ Inhibitors of the cGAS‐STING pathway could serve as potential drug targets for treating hepatic I/R injury.

The cGAS‐STING pathway is crucial in innate immunity, detecting double‐stranded DNA (dsDNA) and initiating robust type I interferon and inflammatory responses.^7^, ^8^ Our prior research indicates a significant association between cGAS‐STING pathway activation and NAFLD, liver lipid drop disturbance, as well as liver cancer immunotherapy.^9^, ^10^, ^11^, ^12^ The cGAS‐STING signaling is precisely manipulated to maintain immune homeostasis and prevent excessive inflammation. Our recent studies indicate significant upregulation of cGAS and STING expressions and activation of the cGAS/STING pathway in I/R injury. Targeting the cGAS/STING pathway offers therapeutic potential for liver I/R injury, with its inhibition markedly enhancing recovery.^13^

Dimethyl fumarate (DMF) has been approved for decades to treat multiple sclerosis or psoriasis because of its immunomodulatory function.^14^ As a fumarate derivative, a key Krebs cycle intermediate, DMF is a potent electrophile that can succinate KEAP1, GAPDH, and GSDMD, thereby inhibiting inflammation and pyroptosis.^15^, ^16^, ^17^ DMF disrupts the interaction of IRAK4 and MyD88 to decrease IFN‐α and inflammatory cytokines release in lasmacytoid dendritic cells.^18^ Another study finds that DMF suppresses LPS‐induced IFN‐β production in macrophages and reduces inflammation‐related coagulation during SARS‐CoV‐2 infection.^19^ However, it is still unclear whether DMF manipulates the cGAS‐STING signaling.

In this study, our findings indicate DMF strongly inhibits the activation of innate immunity in cGAS‐STING pathway by cGAS ligands (mitochondrial DNA [mtDNA], HTDNA, and HSV‐1) or STING agonists (cyclic GMP‐AMP [cGAMP] and diABZI). We evaluated DMF's function on hepatic I/R injury and discovered it inhibits liver enzyme upregulation, cell death, and inflammation activation via a STING‐dependent pathway. Our findings indicate that DMF primarily inhibits the interaction of STING and the downstream molecules TBK1 and IRF3. These findings indicate that DMF mitigates liver I/R injury by inhibiting the cGAS‐STING pathway, revealing its potential function as a therapeutic agent for this condition.

## 2 RESULTS

### 2.1 DMF facilitates HSV‐1 infection through inhibiting type I IFNs

**FIGURE 1:**
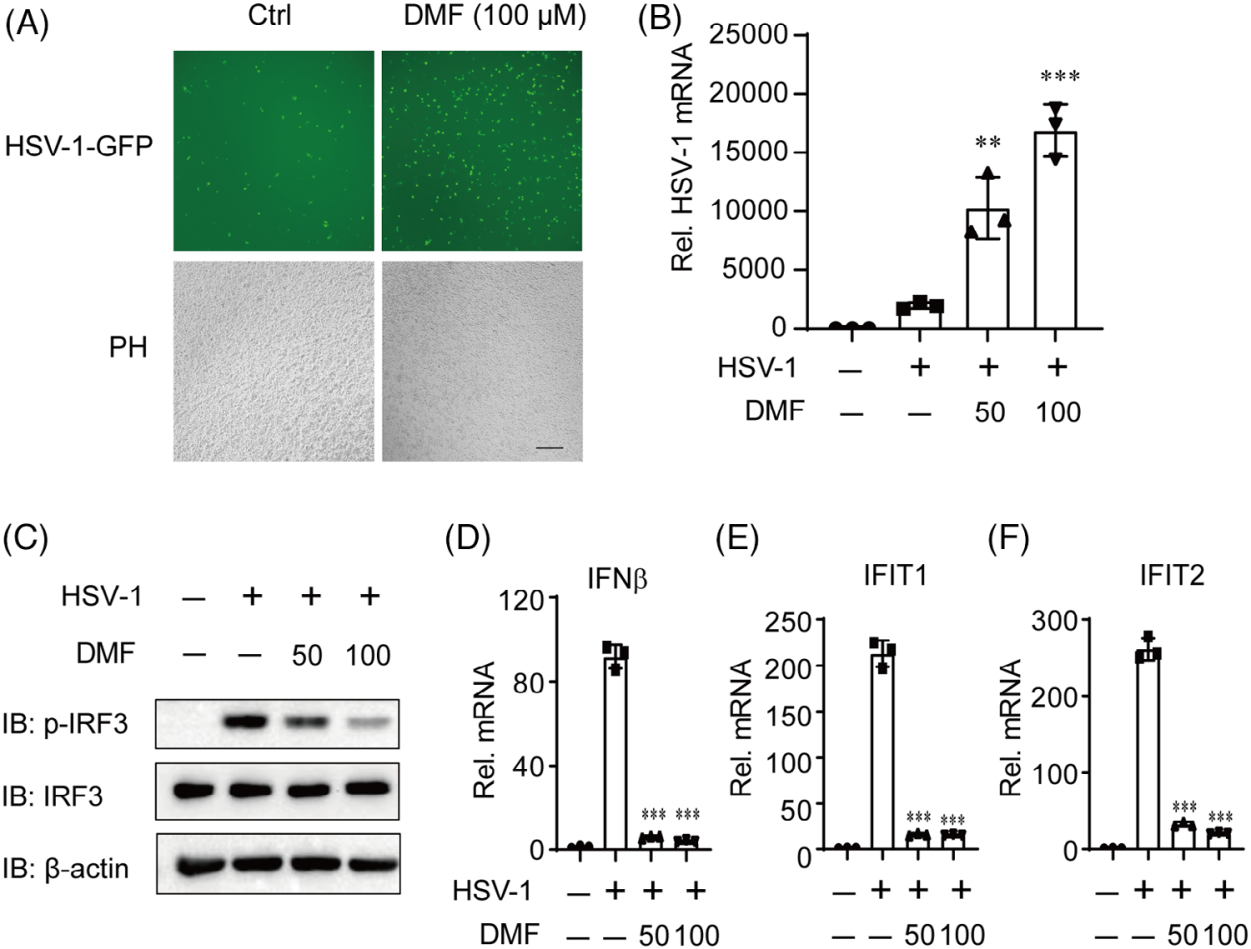
Dimethyl fumarate (DMF) facilitates HSV‐1 infection by inhibiting type I IFNs. (A and B) HeLa cells were infected with HSV‐1‐eGFP for 12 h with or without pre‐treated with DMF (50 or 100 µM) for 12 h. Viral infection was analyzed by fluorescence microscopy (A) and quantitative reverse transcription polymerase chain reaction (qRT‐PCR) (B). Scale bar, 250 µm. (C–F) THP‐1 cells were exposed to HSV‐1‐eGFP for 12 h with or without pre‐treated with DMF (50 or 100 µM) for 12 h. 18s RNA served as the loading control for every real‐time PCR.

DMF, an FDA‐approved immune‐regulatory drug, exhibits significant anti‐inflammatory properties and enhances certain oncolytic virus infections.^20^, ^21^ We exposed HeLa cells to HSV‐1‐GFP (green fluorescent protein) for 12 h and found that DMF can significantly promote the infection of DNA virus HSV‐1 (Figure 1A,B). The host cell's cGAS receptor is crucial for inhibiting HSV‐1 infection and replication by detecting HSV‐1 DNA and activating type I interferon by STING pathway. THP‐1 cells were similarly treated, and immunoblot and qPCR results indicated that DMF significantly inhibited HSV‐1 activated p‐IRF3, type I interferon, and ISG genes (Figure 1C–F). These findings indicate that DMF may enhance HSV‐1 infection and suppress type I IFNs, potentially through the inhibition of the cGAS‐STING pathway.

### 2.2 DMF effectively inhibits cGAS‐STING signaling

**FIGURE 2:**
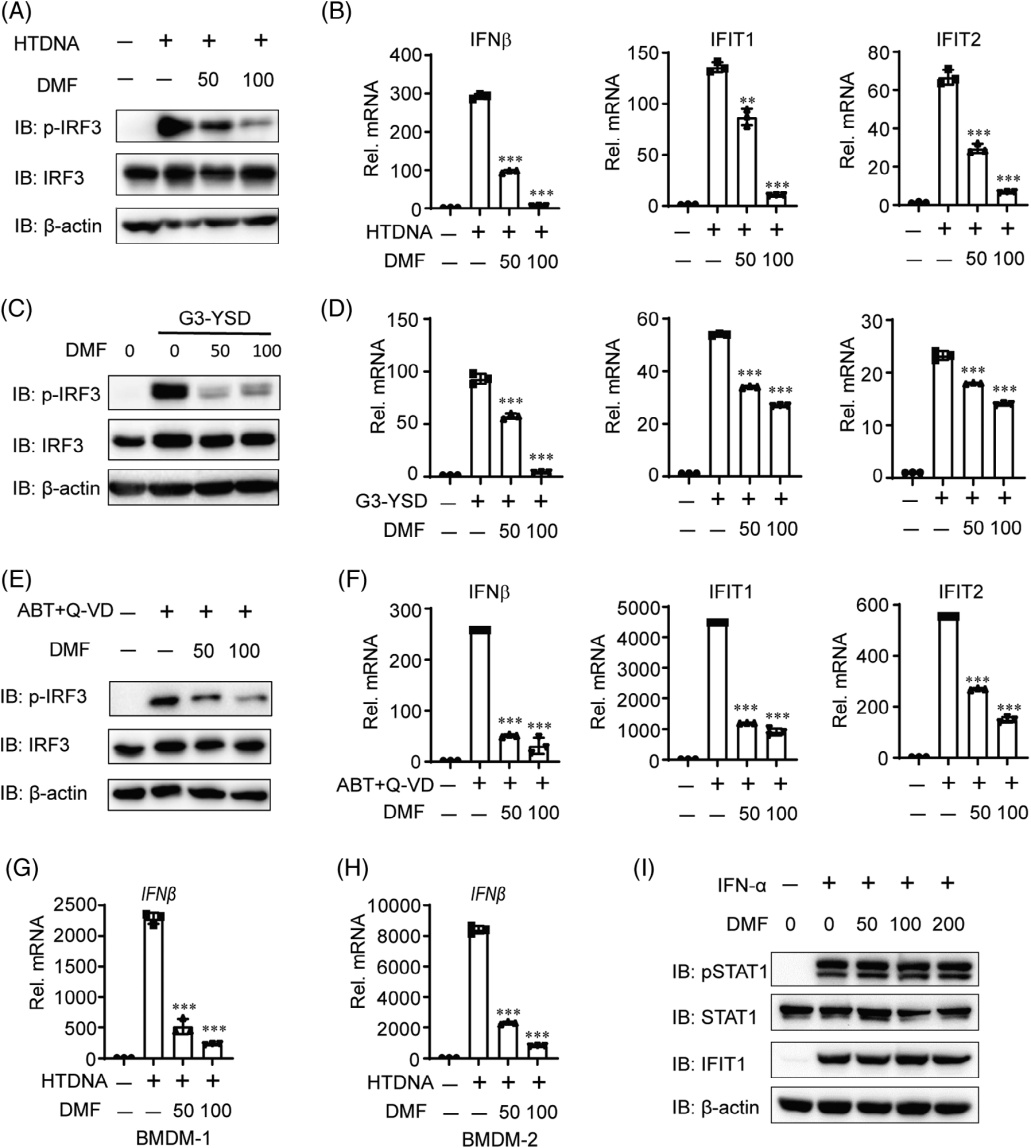
Dimethyl fumarate (DMF) inhibits cGAS‐STING signaling pathway. (A–F) THP‐1 cells were transfected with hering testis DNA (HT‐DNA), G3‐YSD, or treated with ABT737 (10 µM) and Q‐VD‐OPH (10 µM) for 12 h with or without pre‐treated with DMF (50 or 100 µM) for 12 h. (G–H) Bone marrow‐derived macrophages (BMDMs) from mice were pre‐treated with DMF (50 or 100 µM) for 12 h, then transfected with HT‐DNA for 12 h. (I) THP‐1 cells were treated with IFN‐α for 6 h with or without pre‐treated with DMF (50–200 µM) for 12 h. 18s served as the loading control for every real‐time PCR.

To assess the function of DMF on cGAS‐STING signaling, we initially transfected THP‐1 cells with hering testis DNA (HT‐DNA) or G3‐YSD, two known cGAS activators that induces strong cGAS‐STING signaling.^22^ DMF treatment significantly reduced HT‐DNA or G3‐YSD‐induced IRF3 phosphorylation in a dose‐dependent manner (Figure 2A,C). DMF treatment consistently reduced the mRNA expression of IFN‐β, IFIT1, and IFIT2 induced by HT‐DNA or G3‐YSD (Figure 2B,D). Next, we performed dose‐dependent experiments on DMF to determine its function in inhibiting the cGAS‐STING pathway using HTDNA treatment, We found that DMF can inhibit cGAS‐STING activation induced by HTDNA at a dose of at least 20 µm (Figure S1A–D). To verify its role in cGAS‐STING signaling, we treated THP‐1 cells with ABT737 and Q‐VD‐OPH, which induced mtDNA into the cytoplasm and activated cGAS.^23^ DMF treatment suppressed IRF3 activation and decreased the mRNA expression of IFN‐β, IFIT1, and IFIT2 induced by ABT737 and Q‐VD‐OPH, consistent with HT‐DNA and HSV‐1 stimulation (Figure 2E,F). DMF ultimately suppressed HT‐DNA‐induced IFN‐β transcription in mouse bone marrow‐derived macrophages (BMDMs) (Figure 2G,H). In addition, we found that DMF did not significantly inhibit the IFN‐STAT1 pathway (Figure 2I). These findings indicate that DMF inhibits cGAS‐STING signaling.

### 2.3 DMF inhibts cGAS‐STING signaling through an autophagy‐independent pathway

**FIGURE 3:**
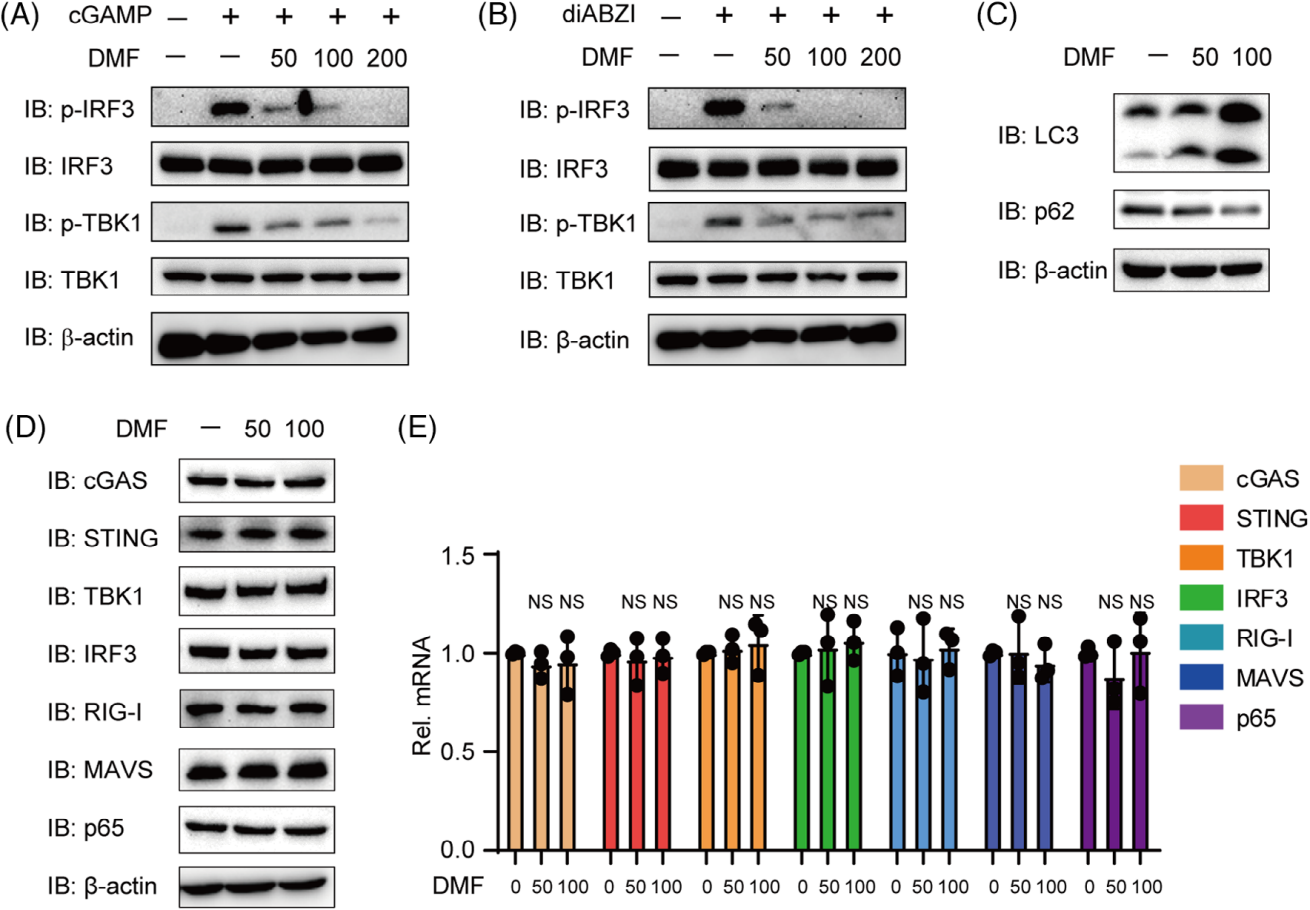
Dimethyl fumarate (DMF) suppresses cGAS‐STING signaling pathway activation through an autophagy‐independent way. (A and B) THP‐1 cells were pre‐treated with DMF for 12 h, then treated with cGAMP (5 µg/mL) for 12 h or diABZI (10 µM) for 2 h. (C–E) THP‐1 cells were treated with DMF (50 or 100 µM) for 24 h, and indicated protein or mRNA was analyzed. 18s served as the loading control.

The experiments demonstrated that DMF effectively inhibits cGAS ligands, including HSV‐1, HT‐DNA, and mtDNA. Next, we aim to examine the impact of DMF on direct STING activation to elucidate its mechanism. We transfected THP‐1 cells with cGAMP and diABZI, two STING robust agonists. DMF treatment significantly inhibited IRF3 and TBK1 phosphorylation and activation under cGAMP or diABZI stimulation (Figure 3A,B). Next, we further explore the mechanism of DMF in cGAS‐STING signaling regulation. In previous study, DMF was found to promote the induction of autophagy.^24^, ^25^ Given that autophagy activation can inhibit the cGAS‐STING pathway,^26^ we hypothesized that DMF may suppress cGAS‐STING signaling by enhancing autophagy and accelerating protein degradation. DMF enhanced autophagy by promoting P62 degradation and increasing LC3‐II expression (Figure 3C). We measured the expression levels of key adaptors and sensors, including cGAS, STING, IRF3, TBK1, RIG‐I, MAVS, and P65. Interestingly, the expression levels of these genes remained unchanged in the presence of DMF (Figure 3D,E). These results indicate that DMF is capable of suppressing STING activation via a mechanism that is independent of autophagy.

### 2.4 DMF inhibits STING signaling independent of Keap1‐NRF2, GAPDH, and GSDMD

**FIGURE 4:**
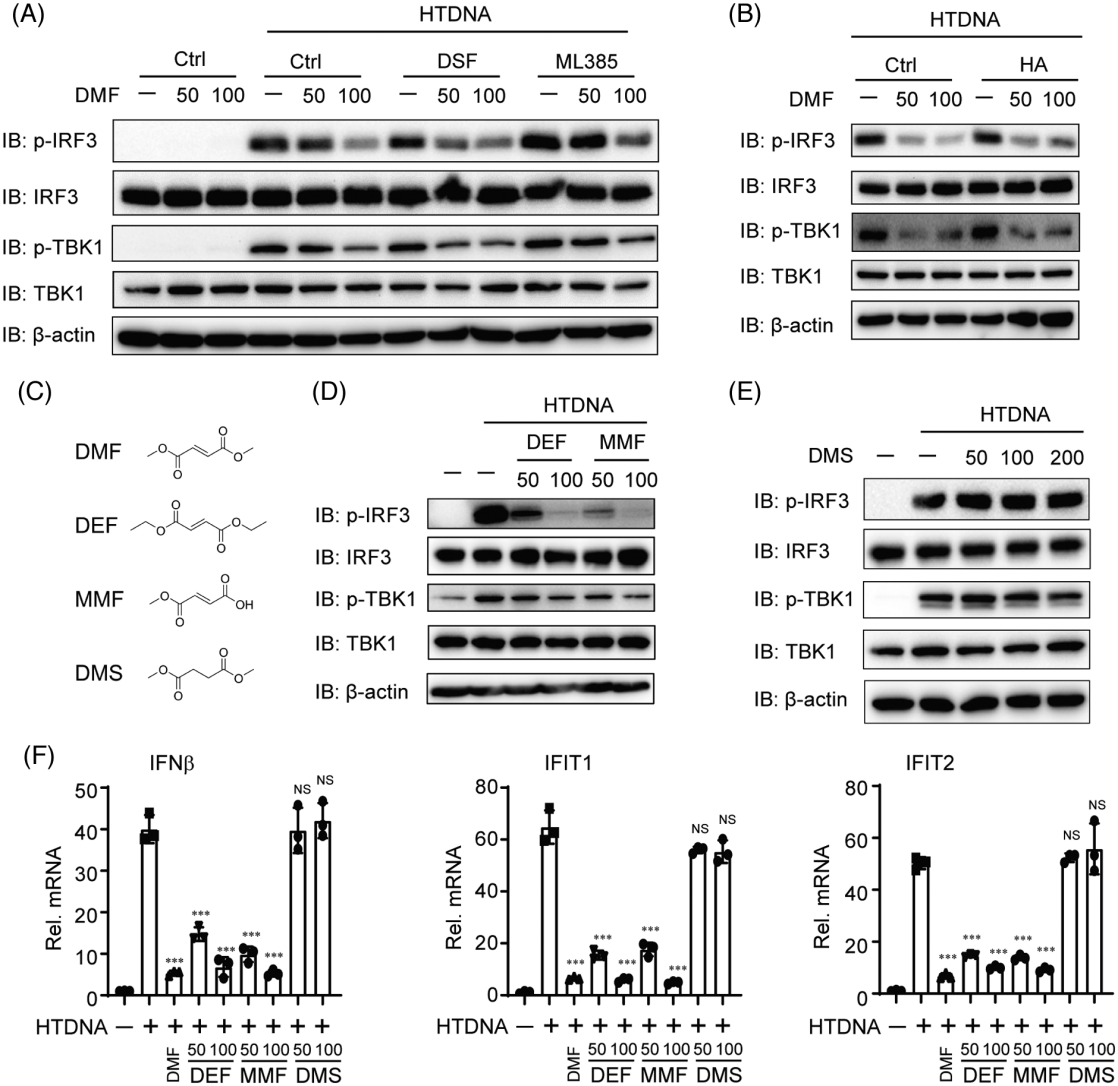
The suppressive role of dimethyl fumarate (DMF) on cGAS‐STING signaling is independent of GSDMD, Keap1‐NRF2, and GAPDH. (A and B) THP‐1 cells were treated for 12 h using HTDNA with or without pre‐treated with DMF (50 and 100 µM), DSF (10 µM), ML385 (10 µM), or HA (10 µM) for 12 h. (C) Chemical structure formula of DMF, diethyl fumarate (DEF), monomethyl fumarate (MMF), and dimethyl succinate (DMS). (D–F) THP‐1 cells were pre‐treated with DEF (50 or 100 µM), MMF (50 or 100 µM), or DMS (50, 100, or 200 µM) for 12 h, subsequently transfected with hering testis DNA (HT‐DNA) for 12 h.

DMF has been identified to directly bind to Keap1, GSDMD, and GAPDH, thereby exhibiting anti‐inflammatory and antioxidant effects through NRF2 activation, and resisting pyroptosis and glycolysis by succinating GSDMD and GAPDH.^27^, ^28^, ^29^ Additionally, cGAS‐STING signaling has crosstalk with NRF2 activation, pyroptosis, and glycolysis.^30^, ^31^ To assess if DMF impacts on cGAS‐STING signaling relies on these three pathways, we treated THP‐1 cells with ML385 (NRF2 inhibitor), disulfiram (GSDMD inhibitor), or HA (GAPDH inhibitor) and found these inhibitors did not counteract the inhibitory effect of DMF on IRF3 and TBK1 phosphorylation induced by HTDNA (Figure 4A,B), indicating that DMF negatively regulated cGAS‐STING signaling was independent of GSDMD, Keap1, and GAPDH.

Fumaric and maleic acid esters (FMAEs) is a big family with anti‐inflammatory effect and neuroprotective effect containing DMF, monomethyl fumarate (MMF), dimethyl succinate (DMS), diethyl fumarate (DEF), and so on^32^, ^33^ (Figure 4C). Therefore, we tested whether other FMAEs similarly inhibit cGAS‐STING signaling. We treated THP‐1 cells with DMF, DEF, MMF, and DMS under HT‐DNA stimulation. Surprisingly, MMF and DEF reduced the phosphorylation of IRF3 and TBK1 activated by HT‐DNA (Figure 4D). Conversely, DMS did not affect the activation of p‐IRF3 and p‐TBK1 (Figure 4E). Finally, we observed that DMF, MMF, and DEF, but not DMS, similarly inhibited the transcription of IFN‐β, IFIT1, and IFIT2 (Figure 4F).

### 2.5 DMF antagonizes cGAS‐STING signaling specifically in the level of STING

**FIGURE 5:**
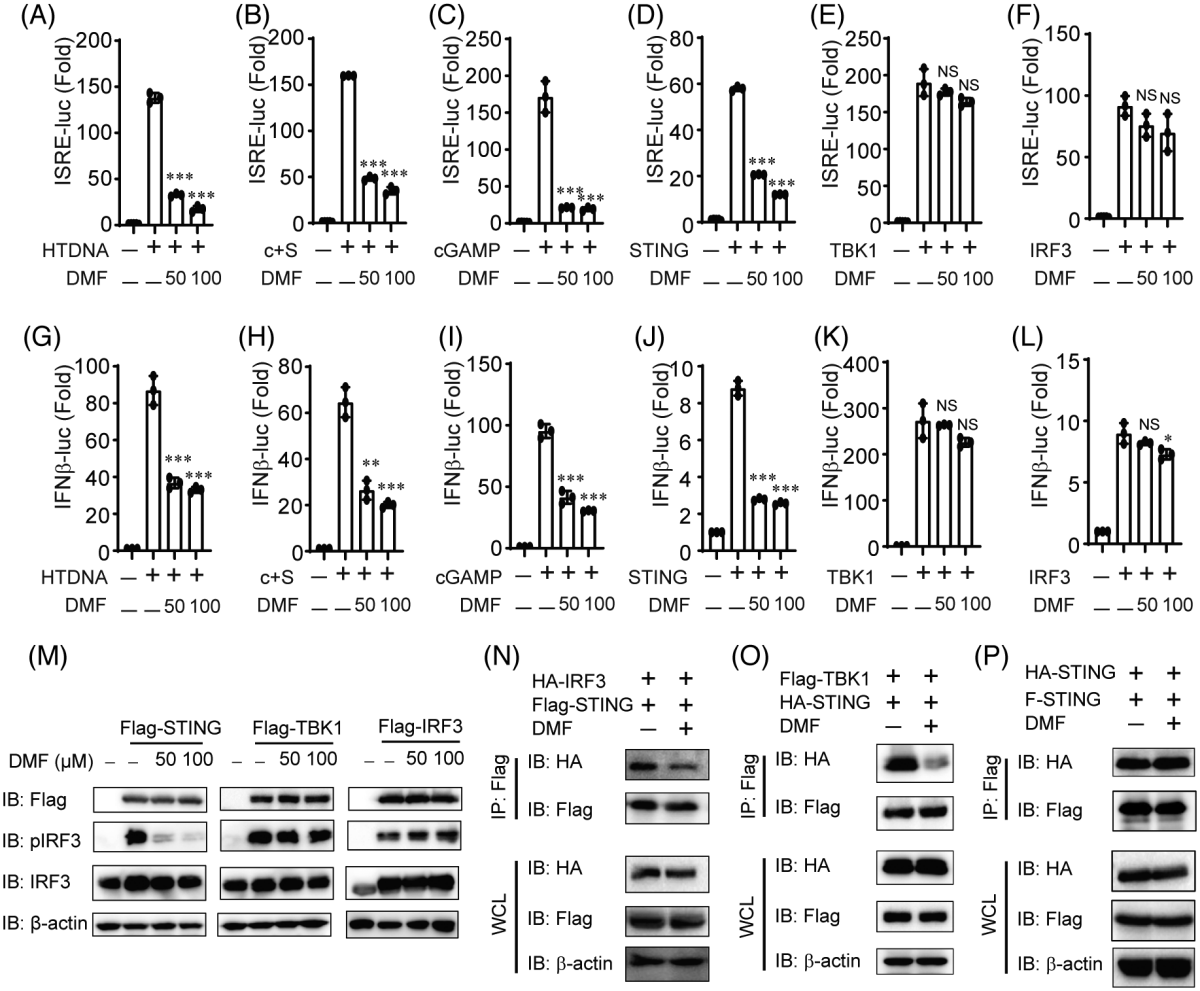
Dimethyl fumarate (DMF) inhibits cGAS‐STING signaling pathway in STING level and suppresses the interaction between STING and TBK1 or IRF3. (A–L) HeLa cells were pretreated with DMF (50 or 100 µM) for 12 h, and hering testis DNA (HT‐DNA), or indicated plasmids were transfected into cells cultured for 24 h. (M) HeLa cells were transfected with indicated plasmids for 12 h with or without pre‐treated with DMF for 12 h. (N–P) HEK293T cells were transfected with indicated plasmids for 12 h, and then treated with DMF (50 µM) for 12 h and co‐immunoprecipitation analysis for the interaction of indicated proteins.

To investigate the mechanism of DMF inhibition on cGAS‐STING signaling, we pretreated HeLa cells with DMF and then transfected them with HT‐DNA, cGAS, STING, cGAMP, TBK1, or IRF3 plasmids, along with ISRE or IFN‐β luciferase reporters. Our findings indicate that DMF significantly inhibits ISRE‐Luc activation induced by HT‐DNA, cGAS‐STING, cGAMP, and STING in a dose‐dependent manner (Figure 5A–D). Likewise, DMF blocked IFN‐β‐Luc activity triggered by HT‐DNA, cGAS‐STING, cGAMP, and STING (Figure 5G–J). DMF did not significantly inhibit ISRE‐Luc or IFN‐β‐Luc activation induced by TBK1 and IRF3 (Figure 5E,F,K,L). In order to confirm this result, we transfected STING, TBK1, or IRF3 plasmids in 293T cells and found that DMF could indeed inhibit p‐IRF3 activated by STING, while it had no significant inhibitory effect on p‐IRF3 activated by TBK1 or IRF3 (Figure 5 M). These results indicated that DMF suppressed cGAS‐STING signaling upstream TBK1 and IRF3, most likely targeting STING.

STING is an important protein for DNA‐triggering type I interferon signaling, which is responsible for recruiting and activating downstream TBK1 and IRF3. We hypothesized that DMF inhibits cGAS‐STING signaling by disrupting the interaction of STING and its downstream proteins. To gain further insight the specific mechanism mediating DMF impacted cGAS‐STING signaling, we overexpressed STING and its several downstream proteins with or without DMF treatment in HEK293T cells. DMF reduced the interaction of STING with TBK1 or IRF3 (Figure 5N–O), however, the combination of STING‐STING was not affected (Figure 5P). These findings suggest that DMF inhibits the interaction of STING with TBK1 and IRF3, blocking downstream pathway activation.

### 2.6 DMF alleviates liver I/R injury dependent on STING

**FIGURE 6:**
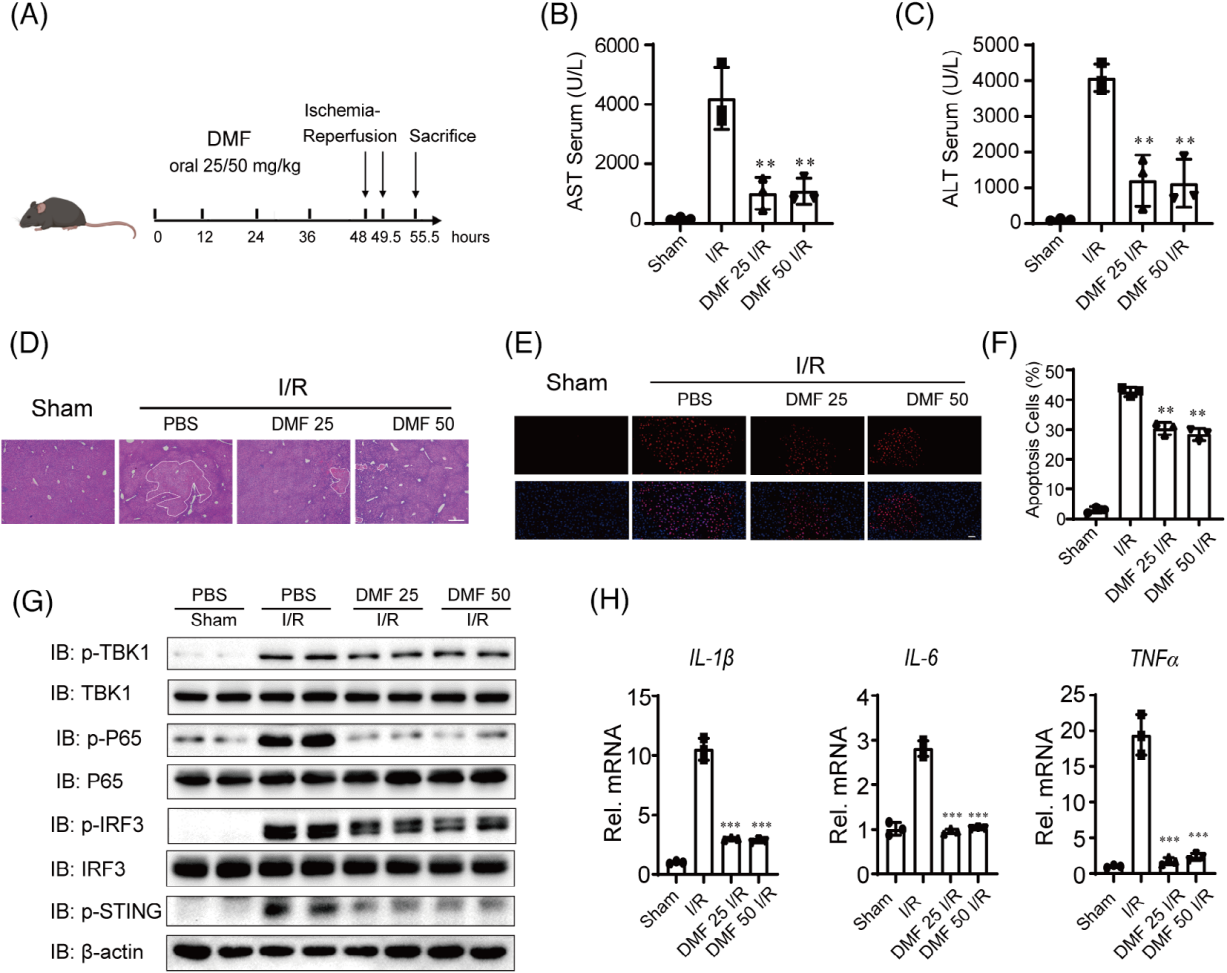
Suitable dose of dimethyl fumarate (DMF) could provide liver protection during hepatic ischemia–reperfusion (I/R) injury. (A) Schematic flowchart of sustained orally given DMF in vivo in hepatic I/R injury mouse model. (B–F) Serum level of ALT/AST (B and C), representative histological H&E‐stained images (D, Scale bar, 200 µm), and TUNEL staining (E and F, scale bar, 50 µm) in liver sections in PBS‐ and DMF‐treated mice at 6 h after hepatic I/R surgery (n = 3 per group). (G) Protein levels of the cGAS‐STING pathway molecules in liver of PBS‐ and DMF‐treated mice at 6 hours after hepatic I/R surgery. β‐actin served as the loading control. (H) Q‐PCR detected the mRNA levels of proinflammatory factors in liver tissue of PBS‐ and DMF‐treated mice at 6 hours after hepatic I/R surgery. 18s RNA served as the loading control for each real‐time PCR.

Hepatic I/R injury is closely linked to cGAS‐STING activation, indicating that inhibitors of the cGAS‐STING pathway might serve as viable therapeutic targets.^13^ Given that DMF inhibits the cGAS‐STING pathway, we assessed its impact on liver I/R injury. The wide‐type (WT) mice were given oral PBS or DMF dissolved in corn oil at a dose of 25 or 50 mg/kg 48, 36, 24 and 12 h before hepatic I/R injury (Figure 6A). Serum level of aminotransferase (ALT)/aspartate aminotransferase (AST), H&E, and TUNEL staining in liver sections showed that DMF had a significant protective effect on liver I/R injury (Figure 6B–F). Meanwhile, we also found that DMF also inhibited the activation of the liver I/R injury‐induced cGAS‐STING pathway and the occurrence of inflammation (Figure 6G,H).

**FIGURE 7:**
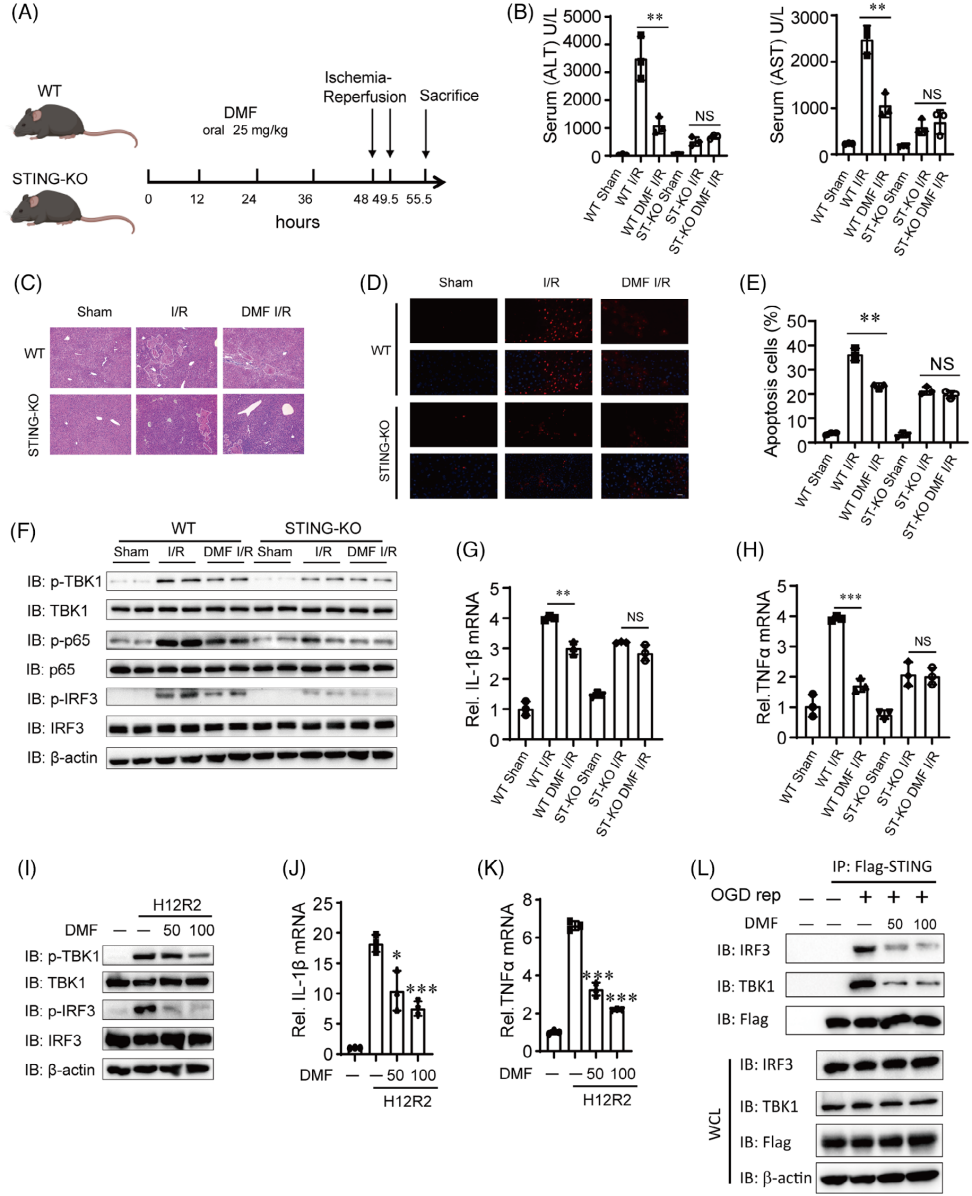
Dimethyl fumarate (DMF) inhibits STING pathway induced by hepatic ischemia–reperfusion (I/R) injury. (A) Schematic flowchart of sustained orally given DMF in vivo in hepatic I/R injury mouse model. (B‐E) Serum level of aminotransferase (ALT)/aspartate aminotransferase (AST) (B), representative histological H&E‐stained images (C, scale bar, 200 µm), and TUNEL staining (D and E, scale bar, 25 µm) in liver sections in PBS‐ and DMF‐treated mice at 6 h after hepatic I/R surgery (*n* = 3 per group). (F) Protein levels of the cGAS‐STING pathway molecules in liver of PBS‐ and DMF‐treated mice at 6 h after hepatic I/R surgery. β‐Actin served as the loading control. (G and H) Real‐time PCR detected the mRNA levels of proinflammatory factors (IL‐1β and TNF‐α) in liver tissue of PBS‐ and DMF‐treated mice at 6 h after hepatic I/R surgery. (I–K) THLE2 cells were treated for 12 h under OGD and 2 h under OGD‐Rep condition with or without pre‐treated with DMF (50 or 100 µM) for 12 h. The phosphorylated TBK1 (p‐TBK1) and phosphorylated IRF3 (p‐IRF3) were analyzed by western blotting (I). The mRNA expression of IL‐1β and TNF‐α was detected by real‐time PCR (J and K). 18s served as the loading control. (L) Flag‐STING‐THLE2 cells underwent OGD‐rep treatment and co‐immunoprecipitation analysis for the interaction of STING‐TBK1 and STING‐IRF3.

Next, we investigated the protective role of DMF against liver I/R injury in STING knockout (KO) mice. The WT and STING‐KO mice were treated in the same way as described above (Figure 7A). DMF pre‐treatment significantly reduced serum ALT and AST activities in WT mice compared to PBS‐treated mice, but this effect was not observed in STING‐KO mice (Figure 7B). DMF reduced the necrotic area and apoptosis during liver I/R injury only in WT mice as well, and DMF‐treated STING‐KO mice had similar necrotic area and apoptosis with PBS‐treated STING‐KO mice (Figure 7C–E).

Next, we further investigated the reasons for the activation of the STING pathway during liver I/R injury. A substantial rise in serum DNA concentrations was noted after liver I/R injury (Figure S2A). Additional verification showed that the increased DNA was predominantly mtDNA rather than nuclear DNA (Figure S2B,C). We found that mtDNA is indeed released during liver I/R injury (Figure S2D), and we further found that hepatocytes but not macrophages significantly release mtDNA (Figure S2E,F), which is consistent with our previous findings.^13^

We propose that the exposed mtDNA activates the cGAS‐STING pathway. In WT mice, the DMF‐treated group showed reduced expression of p‐TBK1, p‐P65, and p‐IRF3 compared to the control model group, whereas no reduction was observed in STING‐KO mice (Figure 7F). The qPCR demonstrated a substantial decrease in IL‐1β and TNF‐α mRNA expression in the liver tissues of DMF‐treated WT mice, whereas no such reduction was observed in STING‐KO mice (Figure 7G,H). Lastly, we also simulated the I/R state through cell oxygen‐glucose deprivation (OGD) and reperfusion experiments. The results showed that DMF alleviated the TBK1 and IRF3 activation and inflammatory factors upregulation (Figure 7I–K). DMF inhibited the co‐immunoprecipitation of STING with IRF3 or TBK1 in Flag‐STING‐THLE2 cells after OGD reperfusion (Figure 7L). These findings suggest that DMF mitigates inflammation during hepatic I/R injury by suppressing STING pathway.

Finally, we assessed the potential tissue damage caused by DMF. We initiated a fresh round of animal studies, adhering to the established dosing and administration protocol for PBS or DMF. Serum biochemical indicators, such as alanine ALT, AST, blood urea nitrogen, and creatinine, for WT mice across all groups were found to be within the normal limits (Figure S3A). Histopathological assessments indicated the absence of significant lesions in the principal organs of WT mice administered DMF (Figure S3B). These results demonstrate that the therapeutic dosage of DMF is safe for mice.

## 3 DISCUSSION

Hepatic I/R injury is a complex condition influenced by numerous signaling pathways and involving a variety of hepatic cell types. Previous research has shown that Kupffer cells and hepatocytes play a crucial role in liver I/R injury.^13^, ^34^ Emerging studies suggest that the cGAS‐STING pathway is intimately linked to hepatic I/R injury and may serve as a promising therapeutic target for its management and prevention. This investigation assesses the effects of DMF on liver I/R injury by probing its suppressive influence on the cGAS‐STING signaling pathway. During liver I/R injury, damaged and dying hepatic cells release mtDNA, which is detected by other liver cells. This detection triggers the cGAS‐STING pathway, which results in inflammation and worsens I/R injury. Pretreatment with DMF inhibits the interaction between STING and its downstream targets TBK1 and IRF3, thereby blocking the STING signaling pathway, decreasing inflammation, and safeguarding the liver. Our findings indicate that although DMF can significantly enhance autophagy, it does not have a significant impact on the protein and mRNA levels of cGAS and STING. Studies have shown that cGAS, STING, and other molecules can be degraded by autophagy, and the way of degradation is dependent on cargo recognition receptors. We believe that DMF can affect autophagy, but it has no effect on the selective autophagy of specific proteins, such as cGAS/STING. This study initially demonstrated DMF's inhibitory effect on the cGAS‐STING pathway, followed by an investigation into its protective effect on liver I/R injury. DMF shows promise as a protective agent against liver I/R injury and excessive inflammation.

**FIGURE 8:**
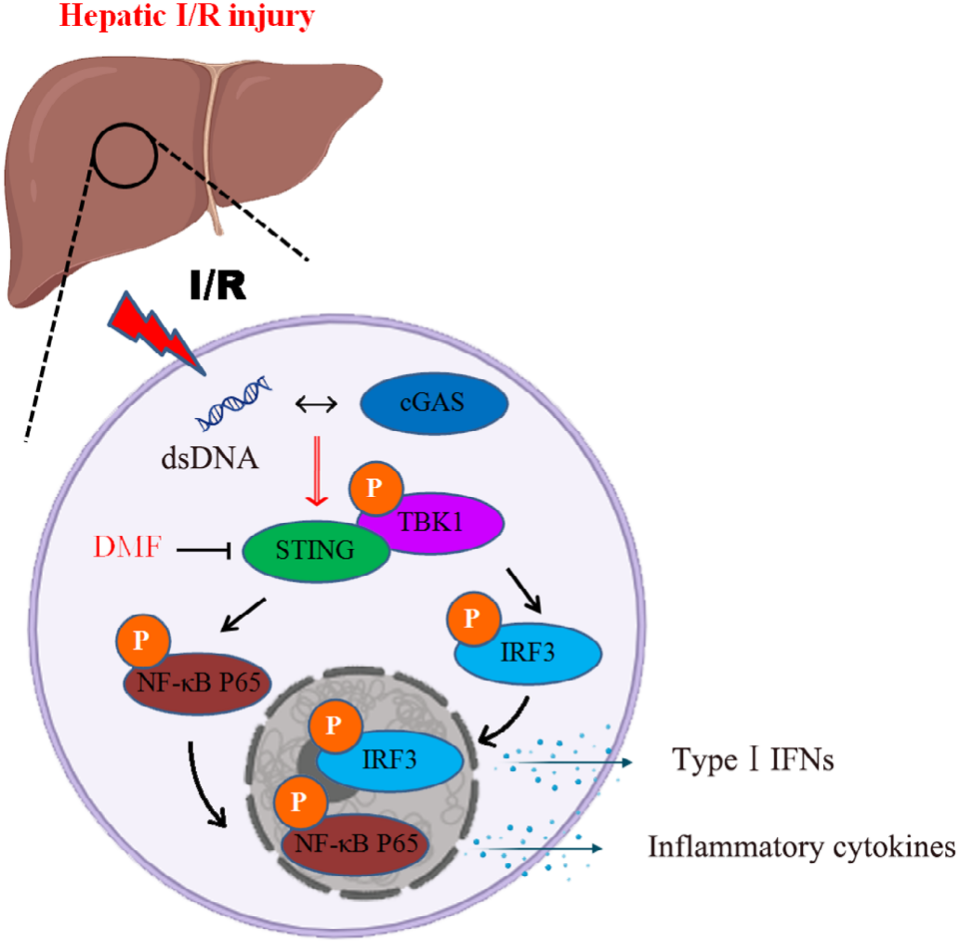
Schematic model of targeting cGAS‐STING by dimethyl fumarate (DMF) to regulate hepatic ischemia–reperfusion (I/R) injury. During hepatic I/R injury, increased mitochondrial DNA (mtDNA) triggered the activation of cGAS‐STING signaling. DMF can inhibit the interaction between STING and downstream TBK1 or IRF3, block the activation of STING, inhibit the occurrence of inflammation, and protect liver I/R injury.

The cGAS‐STING pathway plays a pivotal role in innate immunity by detecting aberrant cytoplasmic dsDNA and initiating an immune response. This pathway is subject to stringent regulation by multiple mechanisms, which is vital for preserving immune homeostasis. Current scientific efforts are concentrated on understanding the regulatory mechanisms of the cGAS‐STING signaling and on the development of potent modulators, including both agonists and inhibitors, for this pathway. Our group is dedicated to investigating the regulation of the cGAS‐STING pathway and its association with liver diseases. Hyperbaric oxygen promotes cGAS‐STING activation, aiding liver cancer immunotherapy.^11^ We demonstrated that TRIM14 influences the selective autophagic degradation of cGAS by modulating its ubiquitination.^26^ Our study indicates that HNF1A targets TBK1 to inhibit the lipid droplet‐activated cGAS‐STING pathway, while STING regulates mTORC1 activation, influencing the autophagic degradation of lipid droplets.^9^, ^10^, ^12^ Furthermore, low doses of sorafenib can inhibit cGAS‐STING activation, suggesting its potential as a treatment for STING overactivation.^35^ This research demonstrates that DMF efficiently suppresses STING activation and the subsequent innate immune reactions by hindering the interaction between STING and TBK1 as well as IRF3, which in turn interrupts the cGAS‐STING signaling cascade. We confirmed that DMF mitigated liver injury by inhibiting STING in a liver I/R injury mouse model (Figure 8). This indicates that DMF can serve as a potential clinical drug for treating diseases caused by overactivation of STING signaling.

DMF is an effective immunosuppressive drug for treating multiple sclerosis. Although previous studies have found that DMF is enable to decrease the production of type I interferon and inflammatory response,^20^, ^21^ whether DMF regulates cGAS‐STING signaling is unclear. Our study confirmed that DMF effectively suppressed cGAS‐STING signaling by hindering the assembly of STING bodies. This effect is independent of several reported DMF targets, including Keap1‐NRF2, GAPDH, and GSDMD. We note that MMF is a hydrolysis product of DMF following oral administration in vivo. DMF, MMF, and DEF share a similar α,β‐unsaturated carbon structure, enabling them to exhibit electrophilic properties and react with cysteine residues in proteins to form S‐(2‐succino)‐cysteine via Michael addition.^18^, ^36^ Conversely, DMS lacks of this functional structure (Figure 4C). Therefore, we infer that the α,β‐unsaturated carbon may undergo Michael addition reaction with a certain protein and modify it. Due to DMF's inhibition of STING transport and activation, we hypothesize that DMF may alter STING and impact its transport function, warranting further experimental validation.

A previous study revealed that DMF attenuates oxidative stress and inflammation while enhancing antioxidant capacity in rats subjected to hepatic I/R injury.^37^ The current study's experiments on the pharmacological protection of hepatic I/R injury in WT mice using DMF mirrored the symptoms previously noted in rats. DMF failed to provide protection against liver I/R injury in STING‐KO mice. These results imply that the STING signaling pathway might be a potential target through which DMF exerts its protective effects against hepatic I/R injury. Our observations suggest that DMF could potentially be used clinically to alleviate hepatic I/R injury, ultimately benefiting patients. This study found no significant toxic effects of DMF on mice, suggesting its potential for further clinical trials to assess its efficacy and safety in treating hepatic I/R injury. DMF, a small molecule drug approved by the FDA for the treatment of multiple sclerosis, has been utilized in clinical practice for more than 20 years with no significant adverse effects. We believe DMF has a high potential as a candidate for liver I/R injury or other diseases caused by abnormal cGAS‐STING activation. Meanwhile, this study only involved animal experiments without any human experiments. It is unclear whether DMF plays the same role in human liver I/R injury, which is a limitation of this study.

## 4 MATERIALS AND METHODS

### 4.1 Cells

HeLa, THLE2, and HEK293T cell lines were cultivated in Dulbecco's modified Eagle medium (Gibco) supplemented with 10% fetal bovine serum (FBS) at a temperature of 37°C in an atmosphere of 5% CO_2_. THP‐1 and BMDMs were grown in Roswell Park Memorial Institute 1640 medium with 10% FBS. The cells were plated in either 24‐ or 12‐well plates and then transfected with poly(I:C) (2 µg/mL), HT‐DNA (2 µg/mL), or cGAMP (5 µg/mL) using Lipofectamine 3000 (Invitrogen, L3000075) for a duration of 12 h, or with diABZI (10 µM) for 2 h. The HSV‐1 are used to infect HeLa cells as described in our previous publications.^38^

### 4.2 Animals

Male C57BL/6 mice, aged between 8 and 10 weeks, were procured from Bestest Biotechnology located in Zhuhai, Guangdong. Male C57BL/6 mice with a STING gene KO were obtained from the Jackson Laboratory. All the mice were housed and bred under specific pathogen‐free conditions in the animal facility at South China Agricultural University.

### 4.3 Antibodies and reagents

The chemical reagents were used as follows: poly(I:C) from InvivoGen, HT‐DNA from Sigma, 2′3'‐cGAMP from APExBIO, diABZI from Selleck, DMF, MMF, ML385, disulfiram and heptelidic acid from MedChemExpress, and DMS from RHAWN.

The antibodies were used for the immunoblot analyses as follows: anti‐p‐TBK1 antibody (CST, 5483S), anti‐TBK1 antibody (CST, 3031S), anti‐p‐IRF3 antibody (Abcam, ab76493), anti‐IRF3 antibody (Huabio, ET1612‐14), anti‐IFIT1 antibody (CST, 14769S), and anti‐STING antibody (CST, 13647S). Antibodies used in the study include anti‐MAVS (Santa Cruz Biotechnology, sc‐166583), anti‐RIG‐I (Cell Signaling Technology, 3743S), anti‐cGAS (Cell Signaling Technology, 15102S), anti‐p‐P65 (Huabio, S529), anti‐LC3 (Proteintech, 18726‐1‐AP), anti‐p62 (Proteintech, 18420‐1‐AP), anti‐beta‐actin (Santa Cruz Biotechnology, sc‐47778), HRP‐anti‐Flag (Sigma, A8592), anti‐HA Tag (Sigma, 05–904), anti‐rabbit IgG, HRP‐linked (Cell Signaling Technology, 7074, 3677, 36788), and anti‐mouse IgG, HRP‐linked (Cell Signaling Technology, 7076S).

### 4.4 Real‐time PCR analysis

The ChamQ SYBR qPCR Master Mix was employed for the quantitative reverse transcription polymerase chain reaction (qRT‐PCR) analysis. mRNA expression levels were determined at Vazme in Nanjing, China, following the prescribed protocols. The qPCR data were normalized against 18S rRNA expression. The sequences of the primers used are detailed in Table S1.

### 4.5 Expression level of detected DNA

Mouse serum DNA was identified and measured following a previously established protocol.^13^ To begin, the QiAmp DNA extraction kit (Qiagen, catalog number 51306) was utilized to extract DNA from 100 µL aliquots of mouse serum that had been filtered through a 0.2‐µm polyether sulfone membrane (Sartorius, product number VS0171). qRT‐PCR was then conducted to assess the quantities of mitochondrial and nuclear DNA. In these assays, the ND2 gene served as a marker for mitochondrial DNA, while the β‐actin gene was used as a marker for nuclear DNA.

### 4.6 Luciferase and reporter assays

HeLa cells underwent transfection with the Lipofectamine 3000 (Invitrogen) after a 12‐h pre‐treatment with DMF. The transfection included plasmids encoding an ISRE or IFN‐β luciferase reporter and TK‐luc, along with plasmids encoding cGAS and STING, STING, TBK1, IRF3, HTDNA, or cGAMP. The luminescence produced by luciferase was quantified employing the Dual‐Luciferase Reporter Assay System from Promega.

### 4.7 Mouse hepatic I/R injury model establishment

We employed a well‐documented rodent model for 70% hepatic I/R injury.^39^ Under this protocol, mice were first sedated with an intraperitoneal administration of 1% pentobarbital. In the control group, no liver ischemia was induced. For the I/R group, a non‐invasive clamp was applied to the blood vessels and bile duct of the left medial and left lateral lobes of the liver, and this clamp was removed after a 90‐min ischemic period. After a 6‐h reperfusion period, the mice were euthanized, and samples of blood, left medial lobe, and left lateral lobe of the liver were harvested.

### 4.8 Statistical analysis

All calculations for statistical significance were performed using GraphPad Prism software, version 9.0.0. Data are presented as the mean with standard error of the mean. For comparing multiple groups, a one‐way analysis of variance along with a Student's *t*‐test was utilized. A *p*‐value of less than 0.05 was set as the threshold for statistical significance. Levels of statistical significance are indicated as: *p < 0.05, **p < 0.01, ***p < 0.001, ns: no significance.

## AUTHOR CONTRIBUTIONS

Yunfei Qin, Dongbo Qiu, and Baoyu Zhang were responsible for the initial idea and the overall study design. Yi Xiong, Jiawen Chen, Kun Li, Wei Liang, and Jinwen Song carried out the experimental procedures. Yi Xiong, Jiawen Chen, and Kun Li were in charge of data acquisition, analysis, interpretation, and they also prepared the initial draft of the manuscript. Xiusheng Qiu oversaw project administration and provided the necessary resources. Yunfei Qin, Dongbo Qiu, and Baoyu Zhang provided critical review and editing of the manuscript. All contributing authors have reviewed and consented to the publication of the final version of the manuscript.

## CONFLICT OF INTEREST STATEMENT

The authors declare no conflicts of interest.

## ETHICS STATEMENT

This study received approval from the Ethics Committee of South China Agricultural University (ethics approval number 2022d084).

## Supporting information



Supporting Information

## Data Availability

Data sharing of the current study were available from the corresponding authors upon reasonable request.
