# Supplementary material for: Dimethyl fumarate alleviate hepatic ischemia–reperfusion injury through suppressing cGAS‐STING signaling
Source: MedComm (2020). 2025 Jan 28;6(2):e70077. doi: 10.1002/mco2.70077 (PMC11773390; doi:10.1002/mco2.70077)
Supplement: Supplementary file 1 — Supporting Information [file MCO2-6-e70077-s001.docx]

**Dimethyl fumarate** **alleviate hepatic ischemia-reperfusion injury through suppressing cGAS-STING signaling**

**Yi Xiong ^1#^, Jiawen Chen ^1#^, Kun Li ^1,2#^, Wei Liang ^1^, Jinwen Song ^1^, Xiusheng Qiu ^3^, Baoyu Zhang ^4^*, Dongbo Qiu ^3^*, Yunfei Qin ^1,3^***

^1^ Biotherapy Center, The Third Affiliated Hospital, Sun Yat-sen University, Guangzhou, Guangdong 510630, PR China.

^2^ Department of Hepatic Surgery and Liver Transplantation Center, The Third Affiliated Hospital of Sun Yat-sen University, Guangzhou, China.

^3^ Vaccine Research Institute, The Third Affiliated Hospital of Sun Yat-sen University, Sun Yat-sen University, Guangzhou, Guangdong 510630, PR China.

^4^ Neurosurgery Department, The Third Affiliated Hospital of Sun Yat-sen University, Guangzhou, Guangdong 510630, PR China.

**^#^**Yi Xiong, **^#^**Jiawen Chen and **^#^**Kun Li made equal contributions to this study.

***Correspondence**

Baoyu Zhang, E-mail: zhangby28@mail.sysu.edu.cn[;](mailto:keekee77@126.com;)

Dongbo Qiu, E-mail: [qiudb3@mail.sysu.edu.cn;](mailto:qiudb3@mail.sysu.edu.cn;)

Yunfei Qin, E-mail: qinyf6@mail.sysu.edu.cn.

**Figure S1**

**
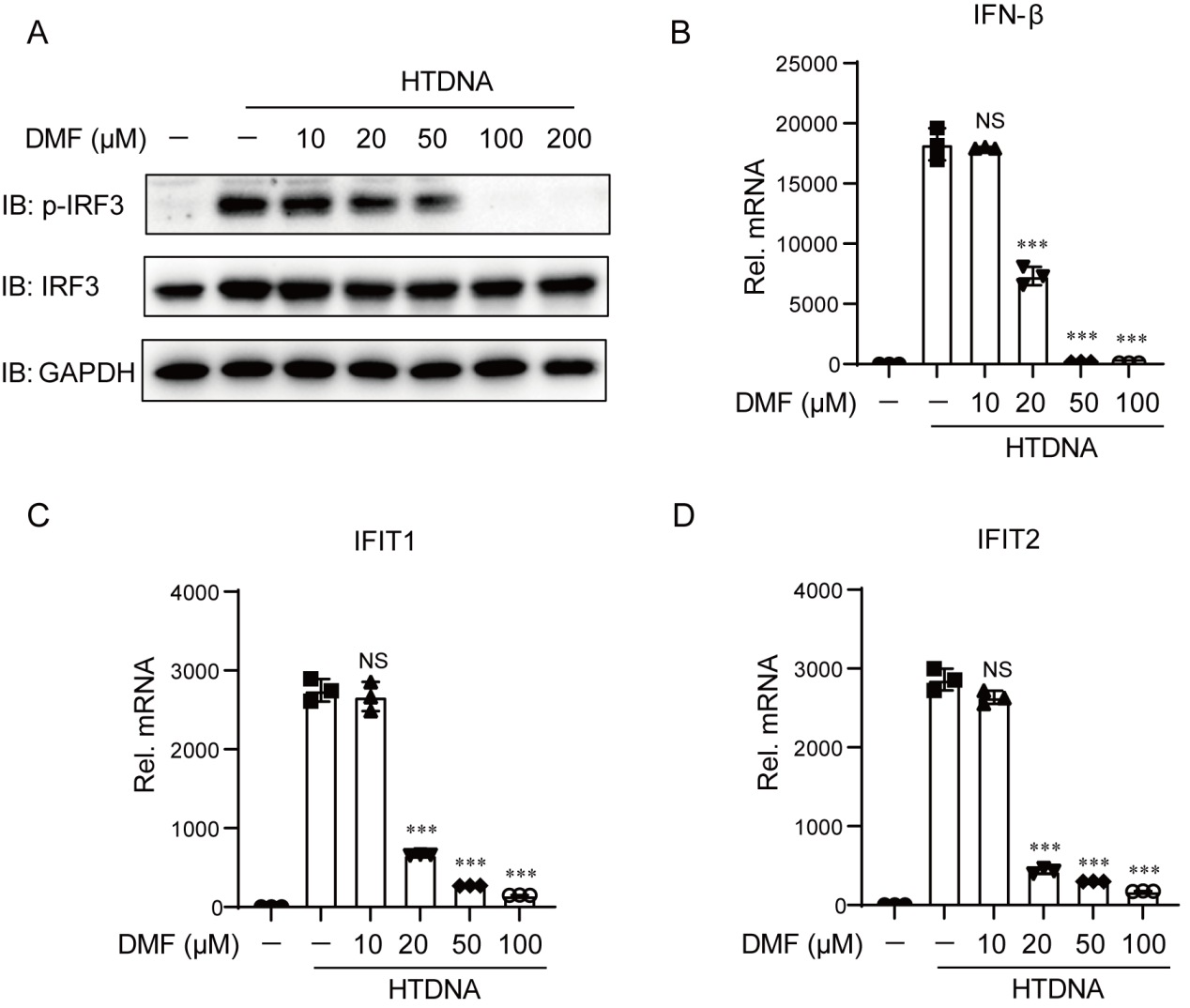
**

**FIG. S1. DMF could inhibit cGAS-STING activation induced by HTDNA at a dose of at least 20 μM.**

**(A-D)** THP-1 cells were subsequently transfected with HT-DNA for 12 hours with or without pre-treated with DMF for 12 hours. Protein levels of p-IRF3/IRF3 or mRNA expression of IFNβ, IFIT1 and IFIT2 were detected by immunoblotting (A) or real-time PCR (B-D). 18s RNA served as the loading control for each real-time PCR. Levels of statistical significance are indicated as: *p < 0.05, **p < 0.01, ***p < 0.001, NS: no significance.

**Figure S2**

**
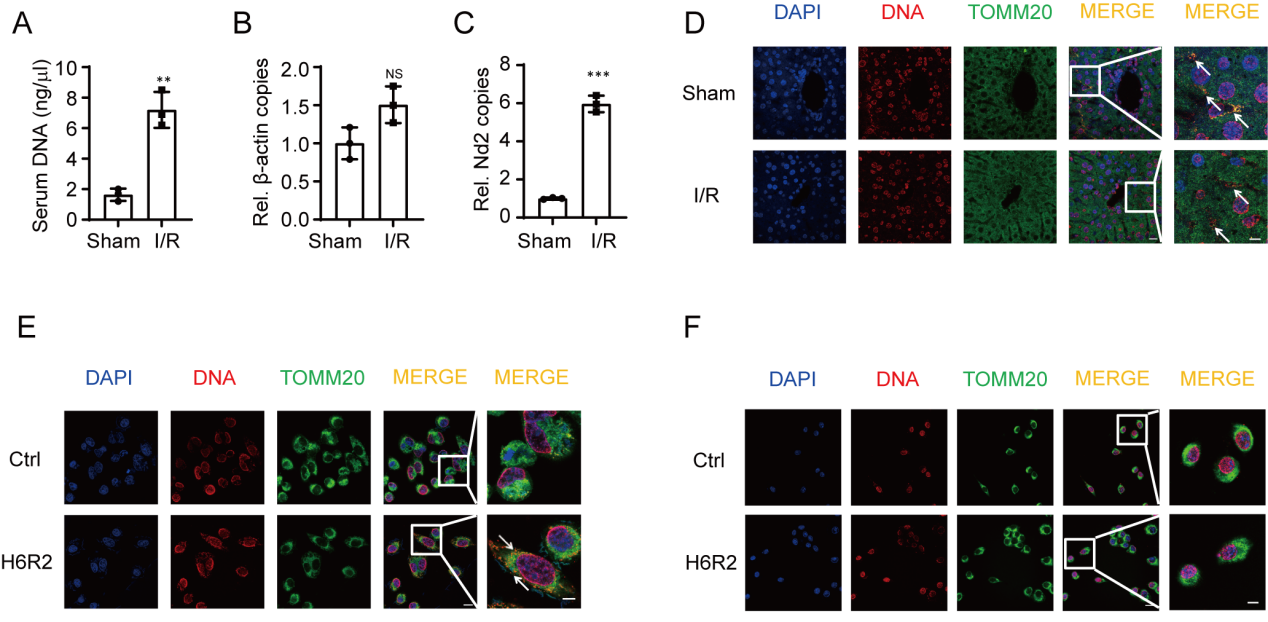
**

**FIG. S2. Leaked mitochondrial DNA increases far more than nuclear DNA during hepatic I/R injury.**

**(A)** Total serum DNA levels of WT mice at 6 hours after hepatic I/R surgery (n = 3 per group). **(B-C)** Relative nuclear DNA copies and mitochondrial DNA detected in serum of WT mice at 6 hours after hepatic I/R surgery (n = 3 per group). Nuclear DNA or mitochondrial DNA present in mouse serum samples was assessed by qPCR using β-actin or Nd2. **(D)** Representative confocal immunostaining images for DNA (red) and TOMM20 (green) in liver sections from WT mice at 6 hours after hepatic I/R surgery. Scale bar, 20 μm. **(E)** Representative confocal immunostaining images in mPHs after OCG-rep treatment. Scale bar, 20μm. **(F)** Representative confocal immunostaining images in liver macrophages after OCG-rep treatment. Scale bar, 20μm. Levels of statistical significance are indicated as: *p < 0.05, **p < 0.01, ***p < 0.001, NS: no significance.

**Figure S3**

**
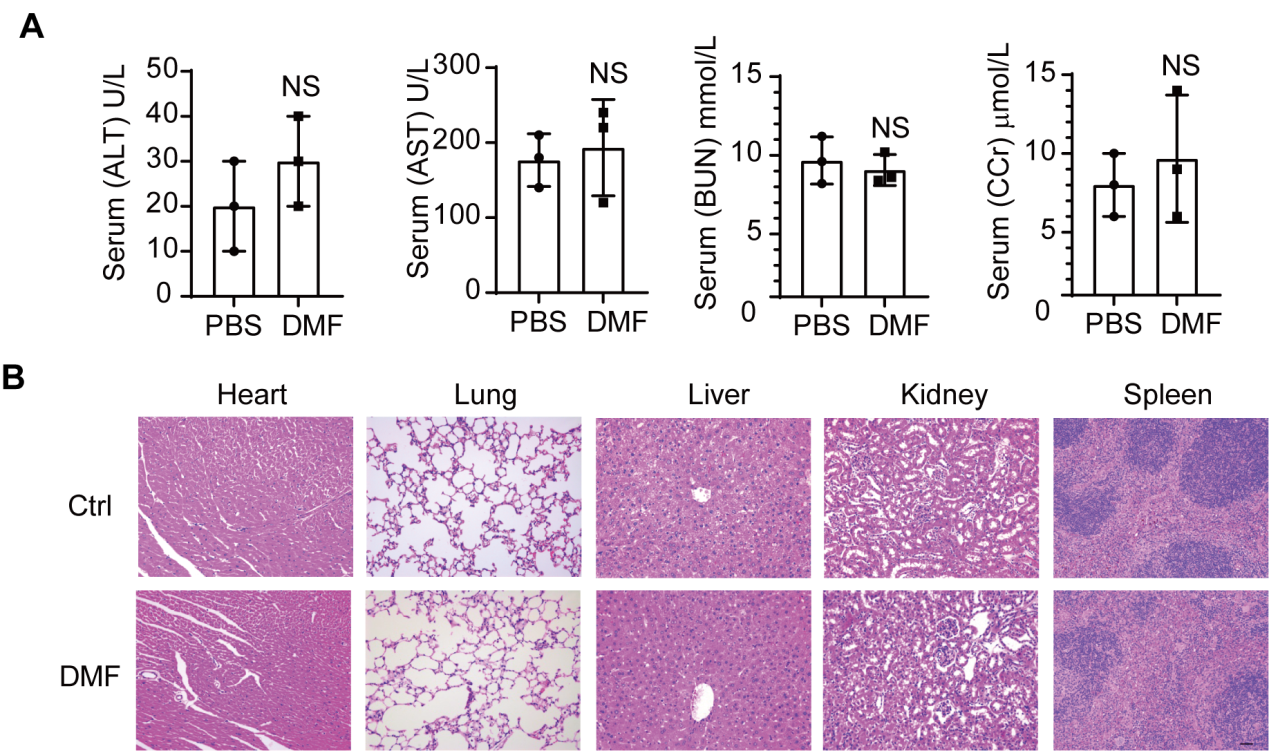
**

**FIG. S3. Suitable dose of DMF promotes liver protection with organ safety. (A)** Serum levels of ALT, AST, BUN, and CCr in WT mice were measured 48 hours after orally given (n = 3 per group). **(B)** H&E staining for heart, lung, liver, kidney and spleen from WT mice in each group (n = 3 per group). Scale bar, 100 μm. Levels of statistical significance are indicated as: *p < 0.05, **p < 0.01, ***p < 0.001, NS: no significance.

**Supplementary Table 1:** PCR primer sequence

| Primers | Species | Sequences |
| --- | --- | --- |
| 18s rRNA | human&mouse | forward primer, 5’- GTAACCCGTTGAACCCCATT -3’ |
|  |  | reverse primer, 5’- CCATCCAATCGGTAGTAGCG -3’ |
| IFNβ | human | forward primer, 5’- ATGACCAACAAGTGTCTCCTCC -3’ |
|  |  | reverse primer, 5’- GGAATCCAAGCAAGTTGTAGCTC -3’ |
| IFIT1 | human | forward primer, 5’- TTGATGACGATGAAATGCCTGA -3’ |
|  |  | reverse primer, 5’- CAGGTCACCAGACTCCTCAC -3’ |
| IFIT2 | human | forward primer, 5’- AAGCACCTCAAAGGGCAAAAC -3’ |
|  |  | reverse primer, 5’- TCGGCCCATGTGATAGTAGAC -3’ |
| RIG-I | human | forward primer, 5’- TGCGAATCAGATCCCAGTGTA -3’ |
|  |  | reverse primer, 5’- TGCCTGTAACTCTATACCCATGT -3’ |
| MAVS | human | forward primer, 5’- CAGGCCGAGCCTATCATCTG -3’ |
|  |  | reverse primer, 5’- GGGCTTTGAGCTAGTTGGCA -3’ |
| cGAS | human | forward primer, 5’- TAACCCTGGCTTTGGAATCAAAA -3’ |
|  |  | reverse primer, 5’- TGGGTACAAGGTAAAATGGCTTT -3’ |
| STING | human | forward primer, 5’- AGCATTACAACAACCTGCTACG -3’ |
|  |  | reverse primer, 5’- GTTGGGGTCAGCCATACTCAG -3’ |
| TBK1 | human | forward primer, 5’- TGCACCCTGATATGTATGAGAGA -3’ |
|  |  | reverse primer, 5’- AAATGGCAGTGATCCAGTAGC -3’ |
| IRF3 | human | forward primer, 5’- AGAGGCTCGTGATGGTCAAG -3’ |
|  |  | reverse primer, 5’- AGGTCCACAGTATTCTCCAGG -3’ |
| P65 | human | forward primer, 5’- CAACCCCTTCCAAGAAGAGCA -3’ |
|  |  | reverse primer, 5’- TTGGGGGCACGATTGTCAAA -3’ |
| IL-1β | human | forward primer, 5’- ATGATGGCTTATTACAGTGGCAA -3’ |
|  |  | reverse primer, 5’- GTCGGAGATTCGTAGCTGGA -3’ |
| TNF-α | human | forward primer, 5’- CCAGACCAAGGTCAACCTCC -3’ |
|  |  | reverse primer, 5’- CAGACTCGGCAAAGTCGAGA -3’ |
| IFNβ | mouse | forward primer, 5’- CAGCTCCAAGAAAGGACGAAC -3’ |
|  |  | reverse primer, 5’- GGCAGTGTAACTCTTCTGCAT -3’ |
| Nd2 | mouse | forward primer, 5’- CCCATTCCACTTCTGATTACC -3’ |
|  |  | reverse primer, 5’- ATGATAGTAGAGTTGA GTAGCG -3’ |
| β-actin | mouse | forward primer, 5’- GATATCGCTGCGCTGGTCG -3’ |
|  |  | reverse primer, 5’- CATTCCCACCATCACACCCT -3’ |
| Il-1β | mouse | forward primer, 5’- TGAAATGCCACCTTTTGACAGTG -3’ |
|  |  | reverse primer, 5’- ATGTGCTGCTGCGAGATTTG -3’ |
| Tnf-α | mouse | forward primer, 5’- GATCGGTCCCCAAAGGGATG -3’ |
|  |  | reverse primer, 5’- TTTGCTACGACGTGGGCTAC -3’ |
| HSV-1 | HSV-1 | forward primer, 5’- ATACCGACGATCTGCGACCT -3’ |
|  |  | reverse primer, 5’- TTATTGCCGTCATAGCGCGG -3’ |
